# Dosimetric characteristics of accelerated partial breast irradiation by interstitial multicatheter brachytherapy with intraoperative free‐hand implantation in the treatment of early breast cancer

**DOI:** 10.1002/acm2.13169

**Published:** 2021-02-24

**Authors:** Chuan Li, Jia‐Fu Lin, Hui Ling Yeh

**Affiliations:** ^1^ Department of Radiation Oncology Taichung Veterans General Hospital Taichung Taiwan; ^2^ Department of Radiation Physics Taichung Veterans General Hospital Taichung Taiwan

**Keywords:** breast cancer, interstitial brachytherapy, accelerated partial breast irradiation

## Abstract

**Introduction:**

The aim of this study is to evaluate the characteristics of the dosimetry and the skin dose of interstitial brachytherapy by the use of the free‐hand implantation technique toward the treatment of early breast cancer.

**Materials & Methods:**

Seventeen patients diagnosed with early breast cancer were selected for the study. The implantation of the catheters for postoperative interstitial brachytherapy was performed using the free‐hand technique. The total tumor dose to the tumor cavity plus 2 cm margin was 3400 cGy, twice daily for 10 fractions in 5 days. The dosage to the target and the organ at risk (OAR) were recorded for analysis. The skin dose of the patient and the phantom were measured with Gafchromic film (EBT3) and the results were compared with the skin dose calculated by the brachytherapy treatment planning system.

**Results:**

The median conformal index is 94% (range 89%–99%), and the median homogeneity index is 71%. The median skin dose measured from the skin of the patients was 20.1% lower than the skin dose calculated from the treatment planning system and consistent with the phantom surface measurement experiment. There were no grade 3 or above acute toxicity recorded.

**Conclusions:**

Interstitial brachytherapy by the use of the free‐hand implantation technique for early breast cancer is feasible and avoids the need for a second surgical intervention. The calculated skin dose was overestimated by at least 20%. The results of this study may help in building a modification model for the prediction of skin toxicity in any future study.

## 1 Introduction

In the era of modern medical practice, the key role of cancer therapy is to give a high cancer control probability, while minimizing the side effects on healthy organs. Conventional whole breast irradiation (WBI) has been a standard adjuvant approach for several decades with regard to early breast cancer after breast conserving surgery (BCS).^1^, ^2^, ^3^ Conventional WBI offers high local control, minimal side effects, and positive cosmetic results.^4^, ^5^ However, conventional WBI after BCS is a time‐consuming treatment that requires a course of 5–6 weeks until completion of the treatment. The difficulties involve transportation, and the adverse effects during radiation therapy may prohibit the patients’ ability to work for their livelihood. Additionally, a significant portion of the normal organs, particularly the lung and heart, are irradiated within the radiation field during conventional WBI.^6^, ^7^ The acute side effects may prohibit the patient from working, while some of the serious late side effects may even cause premature death from heart disease in certain patients.^8^ Accelerated partial breast irradiation (APBI) offers a high dose to the target while reducing risk in a significant portion of normal organs at risk due to the prescribed doses.^9^ APBI increases the quality of life of the patient by reducing the volume of breast irradiated to the tumor cavity plus a 1–2 cm margin, while also shortening the radiation treatment duration to 4–5 days.^10^ The results of recent clinical trials involving APBI revealed that APBI can be an alternative treatment modality to conventional WBI in the treatment of early breast cancer.^11^ APBI has been suggested as one of the treatment options for low‐risk breast cancer patients after BCS in many treatment guidelines.^12^, ^13^ APBI can be performed using interstitial brachytherapy, intraoperative radiotherapy, or external beam irradiation.^14^, ^15^, ^16^

Interstitial brachytherapy for the treatment of early breast cancer has been under investigation for more than 20 years and has been found to be noninferior in terms of local control, overall survival, and disease‐free survival.^11^ Although interstitial brachytherapy is an attractive method of APBI for the treatment of early breast cancer, the high learning curve due to the difficulty of the technique and the lack of recommendations for target delineation and treatment workflow are the reasons for its infrequent utilization in our country. Most of the interstitial brachytherapy cases which have been reported were performed using a sonography‐guided or computed tomography (CT)‐guided multicatheter implantation after the BCS, which in turn required a second operation. The present study reports on our dosimetry analysis of the treatment plans for patients enrolled in interstitial brachytherapy involving intraoperative free‐hand multicatheter implantation at our institution. The intraoperative free‐hand multicatheter implantation technique is a one‐step procedure that allows for the avoidance of a second surgical intervention. Because the skin dose is closely related to the cosmetic results after interstitial brachytherapy, the dosimetry analysis on the comparison of the measured skin dose, the treatment planning system calculated skin doses, and the phantom measured surface doses are also reported in this study. This study was reviewed and approved by our Institutional Review Board (IRB CF17213B). Informed consent has been obtained from all patients in the written form.

## 2 Materials and Methods

**FIG. 1:**
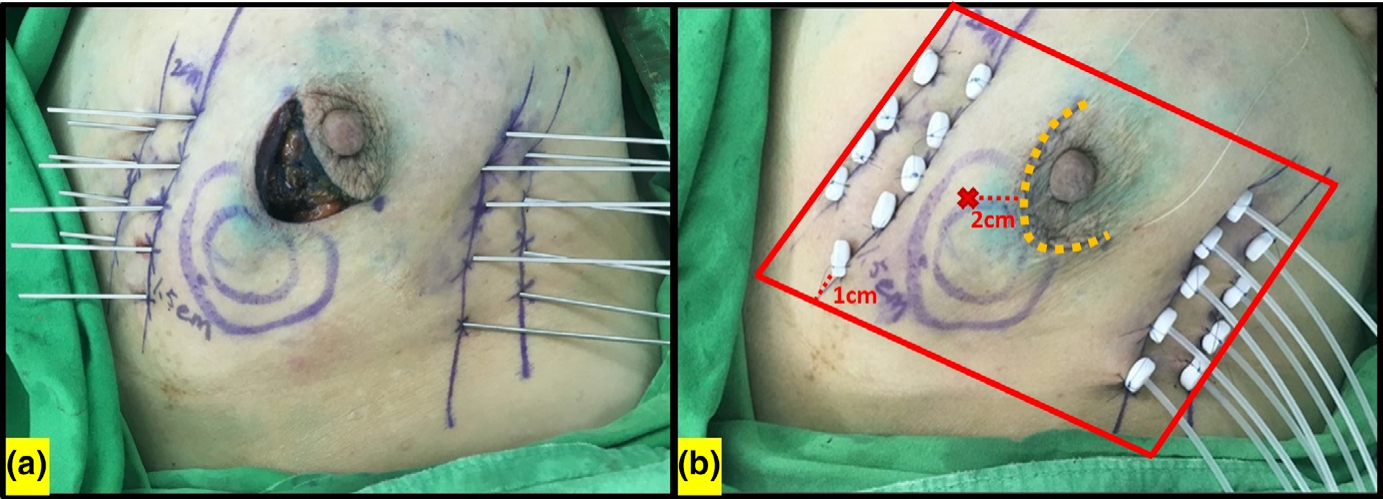
(a) The insertion of stainless‐steel rigid needles after the tumor has been removed. Inner blue circle: skin projections of the tumor bed; outer blue circle: clinical target volume defined as tumor bed with 2 cm margin. (b) The stainless‐steel needles were replaced by plastic catheters and were secured at the skin with buttons. Orange dot line: skin scar; Red Cross: measurement site of the Gafchromic EBT3 film (2 cm for them idles it eoftheskinscar); Red quadrilateral region: measurement region of the skin dose by the treatment planning system.

Seventeen patients diagnosed with early breast cancer who had matched our inclusion criteria for multicatheter high dose rate (HDR) interstitial brachytherapy were selected for the study. The intraoperative implant of the catheters was performed using the open tumor cavity free‐hand technique. For localization of the tumor bed after lumpectomy, surgical findings are incorporated with physician palpation, preoperative sonography, and CT scan. In the surgery, the tumor bed was visualized directly and the extent of the tumor cavity was marked by four surgical clips at the superior, inferior, medial, and lateral sites. The skin projections of the tumor bed [inner blue circle in Fig. 1(a)] and clinical target volume (CTV) were delineated by a marker pen at the joint discretion of the surgeon and the radiation oncologist. CTV is defined as the tumor bed with a 2 cm margin (outer blue circle in [Fig. 1(a)]. After marking the target on the skin surface, the multicatheter insertion was performed through the use of the free‐hand technique. The insertion of stainless‐steel rigid needles (Nucletron Leader Insert Needle, 1.5 × 200 mm) to encompass the tumor bed in 1.5 cm to 2 cm intervals to make one to two planes was dependent on the excision volume in a triangular geometry [Fig. 1(a)]. The stainless‐steel needles were replaced by plastic catheters (Nucletron CT/MR FIT6F,SL,30 cm) which were then secured on both sides with buttons [Fig. 1(b)]. A noncontrast CT scan of the thorax was performed the next day. Before CT scanning, a skin mark was depicted on the skin 2 cm away from the middle site of the surgical scar for the point dose measurement by the Gafchromic film during the treatment [Fig. 1(b)]. The measurement site of skin dose by the treatment planning system (TPS) is a quadrilateral region defined as follows [Fig. 1(b)]: the short sides are the outermost edges of the catheter’s secure buttons, and the long sides paralleled the skin projection of the outermost catheters with 1 cm distance. The superficial boundary is the skin surface. The deep boundary is defined as a 3 mm inner margin from the skin surface. The representative point dose of the involved skin by TPS is D50% (minimum dose to 50% of the volume of the previously defined skin region).

**FIG. 2:**
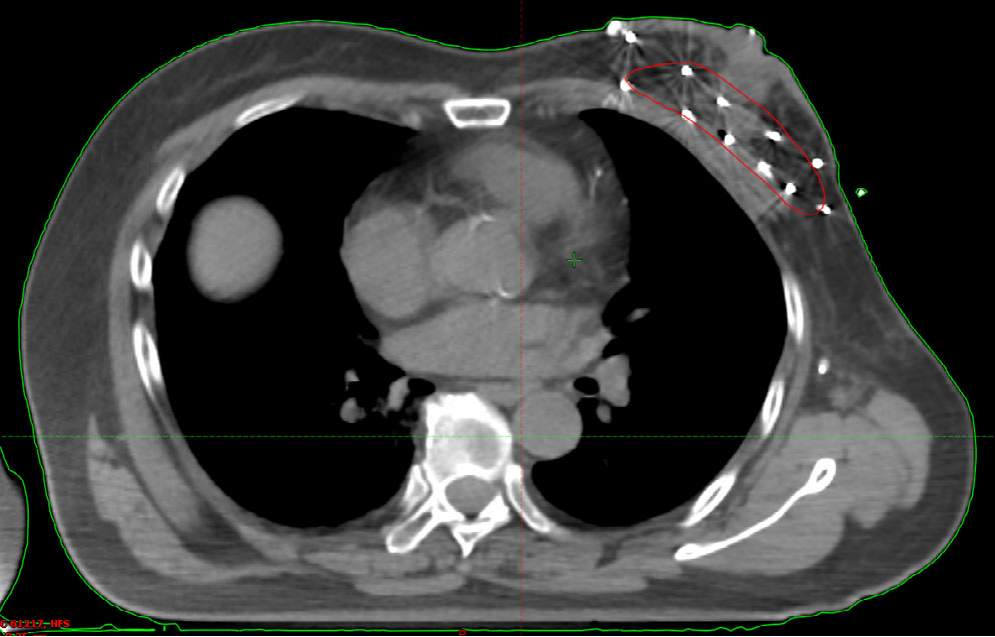
Example of the double‐plane arrangement of the dummy source. Red outline is the clinical target volume (tumor bed with a 2‐cm margin).

The CT scan slice thickness was 2.5 mm and the CT image was acquired with the patient in a supine position after the insertion of the radio‐opaque dummy source (Fig. 2). The CT images were then transferred to our treatment planning system (TPS). The target contouring followed the definition recommended by the International Commission on Radiation Units and Measurements (ICRU) Report 50.^17^ The tumor bed cavity was identified by the surgical clips with or without the seroma. The clinical target volume (CTV) was contoured at a distance of 2 cm from the tumor cavity. The planned target volume (PTV) is defined by the CTV plus a 0.5 cm margin. The ipsilateral breast, the skin, and nearby ribs, as well as the ipsilateral lung and the heart, were also contoured. The treatment planning was designed by the Oncentra Brachy V4.5.3 planning system from the same company. The treatment was initiated on the fourth day after surgery. All patients were treated with the Elekta microSelectron HDR afterloader. A 1 cm × 1 cm Gafchromic EBT3 film was placed on the marked skin area during CT simulation for the skin dose measurement^18^ prior to the initiation of brachytherapy.

The delivered dose was 340 cGy per fraction, twice daily at 6 h apart, for a total of 10 fractions in 5 days, making for a total tumor dose of 3400cGy. The dose constraints were 95% of the target volume covered by 95% of the prescribed dose. The definitions of the involved skin region for dose calculation by TPS are previously described in the first paragraph of the method section [Fig. 1(b)]. The dose constraints for the previously defined skin region was kept at 0.2 cm^3^ volume (D_0.2 cm_^3^) less than 100% of the prescribed dose and 1 cm^3^ volume (D_1 cm_3) less than 90% of the prescribed dose. The involved ribs are defined as the rib volume just beneath the previously defined skin region. The dose constraints for the involved rib was kept at 0.1 cm^3^ volume (D_0.1 cm_^3^) less than 90% of the prescribed dose and 1 cm^3^ volume (D_1 cm_^3^) less than 80% of the prescribed dose. For the ipsilateral nontarget breast, 90% of the volume was covered by less than 10% of the prescribed dose; heart doses were constrained to a mean heart dose less than 8% of the prescribed dose and 0.1 cm^3^ of the heart < 50% of the prescribed dose. The ipsilateral lung was constrained by the mean lung dose < 8% of the prescribed dose and 0.1 cm^3^ of the volume < 60% of the prescribed dose.

The definitions of the key dose–volume parameters for the target are described below. The coverage index (*CI*)^19^ is defined as the fraction of the PTV receiving the prescribed dose. The conformal index (*COIN*)^19^ is calculated using the equation:

(1) $$COIN=\frac{{\mathrm{PTV}}_{\mathrm{PD}}}{V_{\mathrm{PTV}}}\times \frac{{\mathrm{PTV}}_{\mathrm{PD}}}{V_{\mathrm{PD}}},$$

where PTV_PD_ refers to the volume in PTV received the prescribed dose; V_PTV_ refers to the volume of the PTV; V_PD_ refers to the absolute volume irradiated by the prescribed dose.

The homogeneity index (HI)^20^ is calculated using the equation:

(2) $$HI=\frac{V_{\mathrm{PD}}‐V_{1.5xPD}}{V_{\mathrm{PD}}},$$

where V_PD_ refers to the absolute volume irradiated by the prescribed dose and V_1.5xPD_ refers to the volume of the absolute volume irradiated by 1.5x the prescribed dose.

Patient characteristics, dose–volume parameters for the target and OAR, and acute toxicities were recorded and analyzed. D50% of the previously defined skin region by TPS is compared with the point dose by the measurement of EBT3 film on the skin surface. The ratio of skin dose between the measured dose and the calculated dose by TPS is plotted for all 17 patients. The dose difference ratios are plotted and compared between the TPS‐calculated dose and the measured dose from the phantom. The details are described in the following paragraph for the measurement of the EBT3 film on the patient and the phantom.

**FIG. 3:**
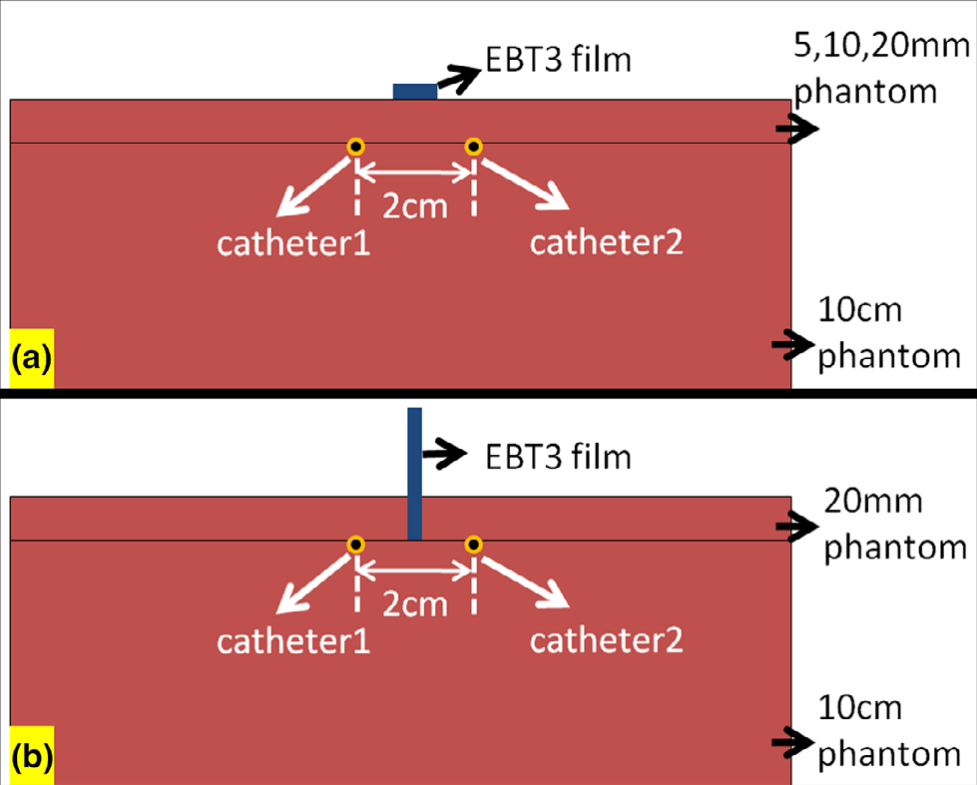
(a) The dose measurement at the phantom surface. (b) The EBT3 film was placed perpendicular to the catheter on the phantom plane.

For the skin dose from the EBT3 film, the measurement site of the skin dose was assigned as the skin surface 2 cm away from the middle section of the surgical scar. To measure the skin dose decay gradient from the catheters, the skin dose measurement of both the patient and the phantom was obtained and compared to the skin dose calculated from the brachytherapy treatment planning system (TPS). The skin dose measurement of the patient and the phantom was performed by using an EBT3 film which was put on the skin surface at the middle section of the surgical scar and 2 cm from the scar to measure the daily skin dose. To avoid the variability involved in measuring different patients, we also conducted control group surface dosage measuring. We planned a therapy plan using two catheters which were placed parallel and 2 cm apart. The dose measurement from the phantom was performed using the point dose measurement at the phantom surface at a distance from the catheter of 5 mm, 10 mm, and 20 mm, as shown in [Fig. 3(a)]. When comparing the TPS calculations and the actual dosage measurements, the dosage tends to be exaggerated. To further confirm that, we set the EBT3 film perpendicular to the catheter on the phantom plane, which was 2 cm away from the surface of the phantom [Fig. 3(b)] in order to execute the experimental measurement plan, and analyze the several dose difference ratios between the measured dosage on the EBT3 film and the TPS dose calculations. The EBT3 films were kept in a dry, dark area at room temperature for at least 24 h before reading. The optical density was found using the Epson Expression 10000XL scanner and then converted into the dosage using a calibration curve by green channel.^21^ All measured doses were expressed as a percentage of the prescribed doses.

## 3 Results

**FIG. 4:**
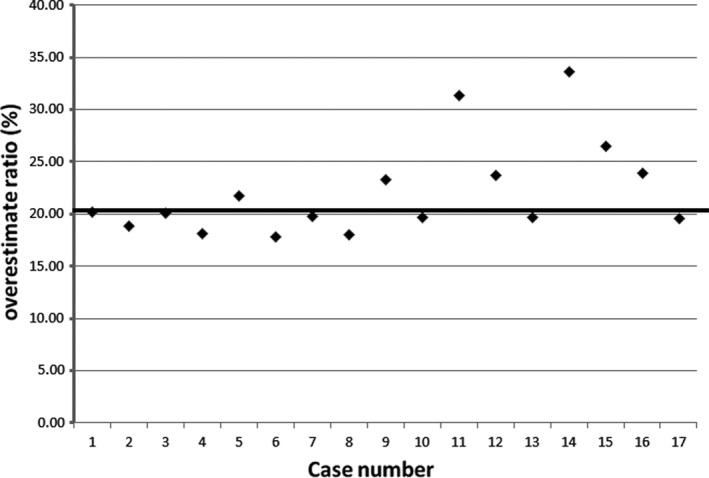
The overestimation ratio of skin dose between the calculated dose by TPS and the measured dose by EBT3 film on the patients. The horizontal line at 20.1% indicates a median percent difference between the calculated dose and the measured dose.

**FIG. 5:**
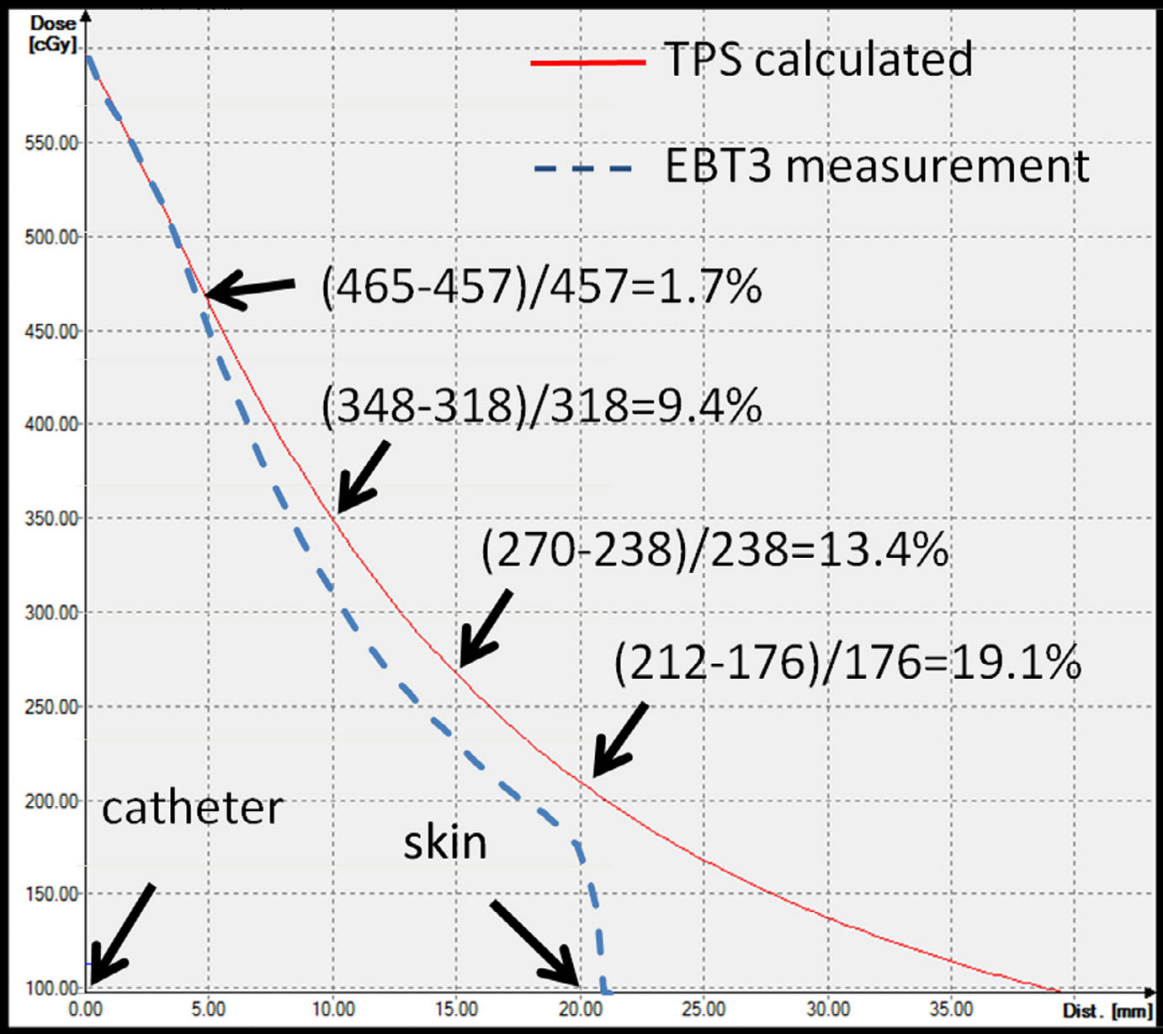
The dose difference ratios between the TPS‐calculated dose and the measured dose by EBT3 film on the phantom.

The intraoperative free‐hand multicatheter implantations were performed smoothly for all of our patients. The patient characteristics are shown in Table 1. Due to the small breast size of Asian women, the use of a double plane arrangement for the catheters was enough to cover the tumor cavity plus 2 cm margins for all of the patients. The dosimetric characteristics of the target parameters and the OAR are shown in Table 2 and Table 3, respectively. The median absolute volume irradiated by the prescribed dose (V_PD_) was 80.48 cc. Only 0.05% of the rib was irradiated by 90% of the prescribed dose (V_90_). The median dose for 1 cm^3^ (D_1 cm_^3^) and 0.1 cm^3^ (D_0.1 cm3_) of the rib was 212.8 cGy and 244.7 cGy, respectively. The median dose for 1 cm^3^ (D_1 cm_3) and 0.2 cm^3^ (D_0.2 cm_^3^) of skin was 226.7 cGy and 258.4 cGy, respectively. The doses for both the ribs and the skin are within the limitations of the constraints (constraints for skin: D_0.2 cm_^3^ < 340 cGy/fraction and D_1 cm_3 < 306 cGy/fraction; constraints for ribs: D_0.1 cm3_ < 306 cGy/fraction and D_1 cm_^3^ < 272 cGy/fraction). Our treatment plans achieved good dose coverage and acceptable dose homogeneity with the median coverage index (CI) of 94.4%, and the median dose homogeneity index (DHI) of 0.71. There was no Grade 3 or above acute toxicity after interstitial brachytherapy. The measured skin dose was overestimated by the TPS.^22^ The median skin point dose measured from the skin surface of the patients was 20.1% lower (range 15%–33%) than the skin doses calculated from the treatment planning system (Fig. 4). The phantom surface point dose difference between the phantom measurement and the treatment planning calculation was 1.7%, 9.4%, and 13.4% when the catheter location was 5 mm, 10 mm, 15 mm below the phantom surface, respectively. The continued increase of the dose difference ratio from the dose measured by the EBT3 film placed perpendicular to the catheter on the phantom plane was overestimated by 19.1%, which is consistent with the point dose measured 2 cm away from the middle site of the surgical scar (Fig. 5).

**TABLE 1:** Patient characteristics.

Age mean (year)	59 (range 45‐75)
Left breast	8 (8/17)
Right breast	9 (9/17)
Histology	
IDC	15 (15/17)
ILC	2 (2/17)
T stage	
T1	13 (13/17)
T2	4 (13/17)
N stage: N0	17 (17/17)
ER +/−	17/0
PR +/−	15/2
HER2 +/−	0/17
Ki‐67 < 14 / >14	14/3
Tumor grade 1/2/3	8/9/0

IDC, invasive ductal carcinoma; ILC, invasive lobular carcinoma; ER, estrogen receptor; PR, progesterone receptor; HER2, human epidermal growth factor receptor 2.

**TABLE 2:** Parameters of target dose distribution.

	Mean	Median	Max	Min	SD
V_PD‐body_ (cc)	82.26	80.48	149.11	36.6	27.76
V_1.5xPD‐body_ (cc)	26.19	26.53	59.57	9.72	11.6
DNR	0.31	0.3	0.4	0.24	0.05
DHI	0.69	0.7	0.76	0.6	0.05
V_PTV_ (cc)	72.39	73.34	125.04	32.3	26.09
V_100_ (cc)	64.86	66.26	115.99	27.5	23.64
V_150_ (cc)	21.06	21	51.11	6.2	10.51
V_200_ (cc)	8.35	8.3	19.86	3	3.84
OI	8.22	8.3	19.86	4.5	3.89
CI	0.94	0.94	0.98	0.86	0.03
COIN	0.7	0.74	0.76	0.52	0.07
D_95_ (%)	93.85	94.3	99.6	85.7	4.05

V_PD‐body_, absolute volume irradiated by the prescribed dose in the whole body; V_1.5xPD‐body_, absolute volume irradiated by the 1.5x prescribed dose in the whole body; DNR, dose non‐uniformity ratio = V_1.5xPD_ /V_PD_; DHI, dose homogeneity index = (V_PD_ –V_1.5xPD_)/V_PD_; V_PTV_, volume of the PTV; V_XX_, absolute volume receiving xx% of the prescribed dose; OI, overdose volume index = V_200_/V_PTV_; CI, coverage index, the fraction of the PTV receiving the prescribed dose; COIN, conformal index = PTV_PD_/ V_PTV_ X PTV_PD_ / V_PD_; D_95_, Percentage of organ receiving 95% of the prescribed dose.

**TABLE 3:** Parameters of OAR dose distribution.

	Mean	Median	Max	Min	SD
Ipsi‐Lung mean dose (cGy/fr)	16.29	15.6	22.1	8.8	3.96
Ipsi‐Lung D_0.1 cm_^3^ (cGy/fr)	170.1	187.8	222.9	83.3	41.38
Heart mean dose (cGy/fr)	11.53	8	26.5	4.1	7.57
Heart D_0.1 cm_^3^ (cGy/fr)	75.34	54.3	149.3	40.5	39.26
Non‐target breast V_90_ (%)	3.84	3.8	9.8	1.1	2.16
Rib V_80_ (cc)	0.05	0	0.24	0	0.11
Rib V_90_ (cc)	0.01	0.05	0.13	0	0.03
Rib D_1 cm_^3^ (cGy/fr)	191.89	212.8	245.2	99.5	45.83
Rib D_0.1 cm_^3^ (cGy/fr)	225.97	244.7	276.7	115.2	56.57
Skin V_100_ (cc)	0.04	0	0.23	0	0.07
SkinV_90_ (cc)	0.09	0	0.58	0	0.16
Skin D_1 cm_^3^ (cGy/fr)	231.02	226.7	369.7	162.9	47.16
Skin D_0.2 cm_^3^ (cGy/fr)	257.89	258.4	344.6	173.4	49.57

OAR, organ at risk; Ipsi‐Lung, ipsilateral lung; cGy/fr, cGy per fraction; V_XX_, absolute volume receiving xx% of the prescribed dose; D_XXcm_^3^, absolute dose given to exposed xxcm^3^ of organ.

## 4 Discussion

Interstitial multicatheter brachytherapy is a less common approach in Asian countries than it is in Western countries. There were only a few studies from Asian countries that reported their treatment results in 2017.^23^, ^24^ All of the reports from Asia have suggested that interstitial multicatheter brachytherapy can be considered as an alternative treatment for early breast cancer after breast conserving surgery in Asian women. The implantation of the catheters can be performed by CT or the ultrasound‐guided method during the second operation under either a closed or open tumor cavity, or performed intraoperatively during the same operation after the tumor has been removed. In our institute, we implement the catheters intraoperatively by the free‐hand method to avoid both a second surgery and the use of anesthesia. The arrangement of the catheters is only done in two planes because the breast size of Asian females is usually smaller than those of their Western counterparts. Currently, with the aid of CT imaging, catheter reconstruction makes the delineation of the target and organ at risk easier to decipher than it had been during the X‐ray era of the past. Our treatment planning was upgraded to use inverse optimization algorithms to provide the requirement of the dose distributions for the dose coverage, dose homogeneity to the target, and the dose to the organ at risk. The parameters calculated from the dose–volume histogram are used for quantitative plan evaluation. The dosimetric analysis of our data shows good coverage of the target and acceptable homogeneity within the target, which is comparable to the dosimetric characters of the sonography‐guided technique or CT‐guided technique implantation. The conformity and homogeneity are not compromised by the use of only two planes of catheters. To reduce the possibility of fat necrosis after interstitial brachytherapy, the median exposure volume of the target to V150% and V200% was 22 cc and 8.3 cc, respectively, which is less than the suggested European Society for Therapeutic Radiology and Oncology (ESTRO) recommendation.^12^

Model‐based dose calculation algorithms (MBDCAs) have been developed for resolving the problems with the tissue heterogeneities.^25^, ^26^ Radioactive source for HDR brachytherapy requires detailed measurement of dosimetric parameters to improve the accuracy of the TPS. Hence, the dosimetric parameters of several radioactive sources are well investigated and compared by Monte Carlo calculations and experimental measurements.^27^, ^28^, ^29^, ^30^, ^31^, ^32^, ^33^ In these radioactive sources, iridium‐192 is the most common radioisotope for HDR brachytherapy due to low average energy of 0.38 MeV and needs less shielding for personnel protection. In previous literature for the dosimetric parameters of iridium‐192, there is a negligible difference (less than 6%) between the dose calculated by TPS and that measured by Gafchromic film.^32^, ^33^ Most brachytherapy treatment planning systems (TPSs) calculate the dose according to the American Association of Physicists in Medicine (AAPM) Task Group No. 43 (TG‐43), which assumes a homogeneous water medium around the brachytherapy sources.^34^, ^35^ TPSs cannot take into account that there is no water around the breast. The overestimation of backscatter results in the overestimation of the exit skin dose.^36^ The breasts of Asian women are generally small, the amount of breast tissue left between the skin and the tumor cavity is relatively little, with nearly no breast tissue located in the back of the tumor cavity. The skin is the main organ related to the cosmetic results of interstitial brachytherapy. It is crucial to determine the skin dose received during interstitial brachytherapy to determine the skin dose–response relationship and set up dose limits for optimal skin sparing. This study has demonstrated that EBT3 film measured the skin point dose to be approximately 20.1% lower (a range of 15%–33% depends on variations in the distance of the catheters in different patients) than the TPS calculated dose, which is comparable to the measured phantom dose. The insertion of the catheter should not be placed within 5 mm of the skin surface of the patient. The overestimation of skin dose by the TPS could be as high as 33% of the actual measured skin dose.

## 5 Conclusion

The dosimetric characteristics of interstitial brachytherapy using the intraoperative free‐hand catheter implantation technique are comparable to the recommendation of some major clinical trials previously performed. This procedure prevents a second surgical intervention. The skin dose is overestimated by TPS around 20%. This result is consistent with the data from the phantom skin dose measurement experiment. The result of this study may help to build a modification model for the prediction of skin toxicity in further dosimetric or clinical studies.

## Ethical approval

This study was reviewed and approved by the Institutional Review Board of Taichung Veterans General Hospital (ethical approval number: IRB CF17213B). Informed consent has been obtained from all patients in the written form.

## Declarations of interest

none.
